# BETWEEN PROLENE^®^, ULTRAPRO^®^ AND BARD SOFT^®^ MESHES WHICH PRESENTS THE BEST PERFORMANCE IN THE REPAIR OF THE ABDOMINAL WALL?

**DOI:** 10.1590/0102-672020210001e1577

**Published:** 2021-06-11

**Authors:** Carlos Alberto Lima UTRABO, Nicolau Gregori CZECZKO, Cesar Roberto BUSATO, Mário Rodrigues MONTEMÓR-NETTO, Leandro LIPINSKI, Osvaldo MALAFAIA

**Affiliations:** 1 School of Medicine, State University of Ponta Grossa, Ponta Grossa, PR, Brazil; 2 Post-Graduate Program in Principles of Surgery, Evangelical Mackenzie Faculty of Paraná, Curitiba, PR, Brazil

**Keywords:** Polypropylene mesh, Abdominal hernia, Immunohistochemistry, Collagen, Tela de polipropileno, Hérnia abdominal, Imunoistoquímica, Colágeno

## Abstract

**Background:**

In the definition of the mesh to be used to correct hernias, porosity, amount of absorbable material and polypropylene should be considered in the different stages of healing process.

**Aim:**

To evaluate the inflammatory reaction in the use of macro and microporous meshes of high and low weight in the repair of defects in the abdominal wall of rats.

**Methods:**

Ninety Wistar rats (*Rattus norvegicus albinus*) were used. The animals were submitted to similar surgical procedures, with lesion of the ventral abdominal wall, maintaining the integrity of the parietal peritoneum and correction using the studied meshes (Prolene^®^, Ultrapro^®^ and Bard Soft^®^). Euthanasia was performed at 30, 60 and 120 days after surgery. The abdominal wall segments were submitted to histological analysis using H&E, Masson’s trichrome, immunohistochemistry, picrosirius red and tensiometric evaluation.

**Results:**

On the 120^th^ day, the tensiometric analysis was superior with Ultrapro^®^ macroporous mesh. The inflammatory process score showed a significant prevalence of subacute process at the beginning and at the end of the study. Microporous meshes showed block encapsulation and in macroporous predominance of filamentous encapsulation.

**Conclusion:**

The Ultrapro^®^ mesh showed better performance than the others in healing process of the abdominal wall.

## Graphical abstract

[Fig f1a]

**Figure f1a:**
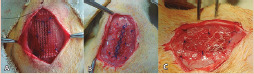
Prolene^®^ Ultrapro^®^ Bard Soft^®^

[Fig f1as]

**Figure f1as:**
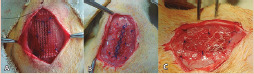
Prolene^®^ Ultrapro^®^ Bard Soft^®^

## RESUMO

**Racional:**

Na definição da tela a ser utilizada na correção das hérnias deve-se considerar a porosidade, quantidade de material absorvível e polipropileno ou inabsorvível nas diversas fases da cicatrização.

**Objetivo:**

Avaliar a reação inflamatória das telas macro e microporosas de alta e baixa gramatura no reparo de defeito da parede abdominal de ratos.

**Método:**

Foram utilizados 90 ratos da raça Wistar (*Rattus norvegicus albinus*). Os animais foram submetidos a procedimentos cirúrgicos semelhantes, com lesão da parede abdominal ventral, mantendo a integridade do peritônio parietal e correção utilizando as telas Prolene^®^, Ultrapro^®^ e Bard Soft^®^. Realizou-se a eutanásia aos 30, 60 e 120 dias de pós-operatório. Os segmentos da parede abdominal foram submetidos à análise histológica com H&E, tricômio de Masson, imunoistoquímica, picrosirius red e análise tensiométrica.

**Resultado:**

No 120º dia a análise tensiométrica mostrou superioridade da tela macroporosa Ultrapro^®^. O escore do processo inflamatório demonstrou prevalência significativa de processo subagudo no início e no final do estudo. As telas microporosas mostraram encapsulamento em bloco e as macroporosas encapsulamento predominantemente filamentar.

**Conclusão:**

A tela Ultrapro^®^ mostrou melhor desempenho em relação às demais na cicatrização da parede abdominal.

## INTRODUCTION

The standard procedure for surgical correction of incisional hernias is using meshes. The most applied material is polypropylene, which causes a rapid acute inflammatory response followed by a chronic persistent foreign body reaction for months and years after the procedure^5^.

Since the first use of the polypropylene mesh and the evolution to the Liechtenstein method as a tension-free method in hernioplasty, the surgical procedure has become common with the use of millions of mesh implanted each year. The polypropylene is the most used in the repair of the abdominal wall, due to its low cost, not biodegradable, and with good tissue incorporation^11^.

The most important parameters for the selection of the meshes are the raw material, structural and mechanical parameters, which must match the physiological conditions. Structural ones, especially porosity, are the most important predictors of good performance in biocompatibility. Those with larger pores exhibit less inflammation and connective tissue, as well as scar bridges, which allow more malleable tissue growth. Synthetic meshes, monofilament and with large pores, show advantages^27^.

Once implanted in the recipient wall, the incorporation with tissue lining should imitate the biomechanical properties of the healthy abdominal wall. Studies have shown that the permanence of the high-weight mesh causes persistent inflammatory reaction at the mesh-tissue interface for months, and even years after implantation^7^.

In order to avoid an exaggerated foreign body reaction, the amount of polypropylene on the mesh or the use of absorbable material that provides initial resistance quickly reabsorbed, reduces local inflammation^19^.

The use of mesh in the repair of hernias induces several complications, such as paresthesia, chronic pain, sperm granuloma, fistula and seroma in about 30-50%. The new designs caused the meshes to be classified as high, medium and low weight, respectively according to density, values ​​above 80 g/m², between 50-80 g/m² or below 50 g/m². Some authors define material with a density below 35 g/m² as ultralight.

The incorporation of biomaterials causes an inflammatory reaction of greater or lesser persistence throughout the life. It has been shown in previous studies that the pore size of surgical meshes impacts the interface of scar formation, and the use of a mesh with reduced material is accompanied by the attenuation of inflammation and fibrosis, and a decrease in the proliferation of apoptotic cells. The polypropylene mesh most commonly used to repair the abdominal wall is polypropylene^21^.

Attempts to reduce the amount of foreign matter have been focused on the design of macropores and on the absorbable and non-absorbable components of the meshes. The pore size is an important factor for new designs, as well as the design of the filaments and their spatial distribution^10^.

The mixed polypropylene/poliglecaprone mesh represents a new member in the group of low weight meshes with large pores. It consists of a monofilament with low weight and large pores - with more than 3 mm - of polypropylene, with the addition of an absorbable Monocryl^®^ component (polyglecaprone 25) that optimize the implant, increase the strength of the wall in the first weeks after the repair. It is fully absorbed without increasing cellularity, inflammation and intense fibrotic reaction between 84 and 140 days.

The partially absorbable mesh, when part of its components undergoes absorption, reduces the amount of foreign material, allowing healing to continue without compromising its biomechanical resistance^16^.

It is known that polypropylene meshes cause early and persistent fibrosis. The reduction of fibrosis is directly provided by the reduction of its weight. The formation of fibrosis bridges is inversely proportional to the pore size^17^.

The appearance of new materials, with larger pores, improved the results, more elasticity and reducing local fibrosis. The use of meshes for the correction of hernias has been intensified since the 1950s, after preliminary studies that showed the validity of using polyethylene in the production of them. The increased resistance of the wall has been proven, but the high porosity of the polypropylene mesh induces an intense inflammatory reaction, with the formation of fibrosis, which reduces the wall elasticity^8^.

Study of meshes composed of low weight biomaterials, using two non-absorbable prostheses - Parietene^®^ and Optilene Elastic^®^ - and two partially absorbable prostheses - Vypro II^®^ and Ultrapro^®^ -, observed that the formation of adhesions on the peritoneal surface of the meshes was significantly less extensive at 90 days post-implant. The partially absorbable meshes showed a higher proportion of macrophages (due to the traces of absorbable material in their structure) than the non-absorbable ones at 90 days, although the differences were not significant. At 90 days the rupture stress was similar in all evaluated meshes. It was concluded that low weight meshes with partially absorbable material may offer advantages over low weight ones non-absorbable, since less foreign material persists in the receiver, allowing improvement in the abdominal wall^3^.

This study aimed to evaluate the healing of a defect produced in the ventral abdominal wall of rats, comparing the repair made with microporous polypropylene (Prolene^®^), macroporous polypropylene (Bard Soft^®^) and polypropylene polyglecaprone (Ultrapro^®^) addressing microscopic and tensiometric reactions of the abdominal wall in the period of 30, 60 and 120 days after the operation.

## METHOD

**FIGURE 1:**
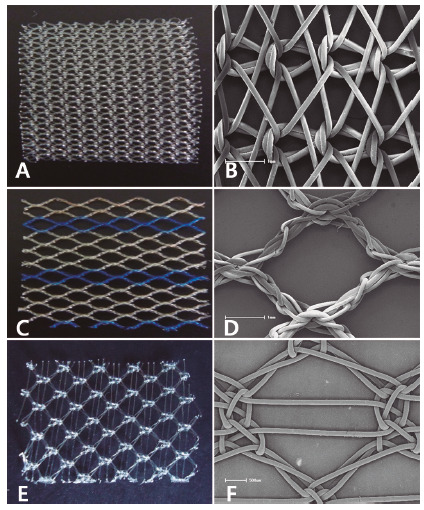
Surgical meshes: A) Prolene®; B) Prolene® (electron microscopy scanning); C) Ultrapro®; D) Ultrapro® (electron microscopy scanning); E) Bard Soft®; F) Bard Soft® (electron microscopy scanning)

The research project was submitted to and approved by the Animal Use Ethics Committee of the State University of Ponta Grossa, Ponta Grossa, PR, Brazil, process nº 037/2014. Ninety male Wistar rats (*Rattus norvegicus albinus*) were used, male, adult young, with three months with weight ranging from 280-300 g. They were divided into three groups of 30 (G1, G2 and G3). All groups underwent similar surgical procedures using three types of mesh: G1, heavy-weight, non-absorbable monofilament mesh with microporous dimensions of approximately 0.9 mm² of polypropylene (Prolene^®^) estimated weight of 100 g/m^2^; G2, monofilament mesh of low density, partially absorbable, with an estimated weight of 28 g/m², macropores of size between 3-4 mm (Ultrapro®) consisting of the combination of equal parts of polypropylene and polyglecaprone; G3, low-density, non-absorbable monofilament, with an estimated weight of 44 g/m² with macropores approximately 6.29 mm² in size, composed of polypropylene (Figure 1).

Each group was divided into three subgroups of 10 rats and evaluated at 30, 60 and 120 days after surgery (Table 1).

**TABLE 1:** Animals by group, mesh used and observation period

Groups	Period	Subgroups
G1 - polypropylene microporous	30 days	G1 30
	60 days	G1 60
	120 days	G1 120
G2 - polypropylene /poliglecaprone	30 days	G2 30
	60 days	G2 60
	120 days	G2 120
G3 - polypropylene macroporous	30 days	G3 30
	60 days	G3 60
	120 days	G3 120

### Surgical meshes

Three types were used: Prolene^®^, Ultrapro^®^ and Bard Soft^®^ (Figure 1).

The rats were subjected to a 12 h preoperative fast and anesthetized with atropine sulfate (0.05 mg/kg of weight) intraperitoneally, and after 10 min, the application of a 2% xylazine hydrochloride mixture (10 mg/kg) body weight) and 10% ketamine hydrochloride (25 mg/kg body weight), that is, 0.2 ml/100 g of solution weight was administered intraperitoneally. When necessary, half the dose was repeated after 20-30 min. They underwent postoperative analgesia with paracetamol orally at a dose of 40 drops for every 500 ml of water offered in the first two days. Euthanasia was performed on the 30^th^, 60^th^ and 120^th^ postoperative day, with a macroscopic evaluation of the surgical wound and the peritoneal cavity.

### Surgical procedure

**FIGURE 2:**
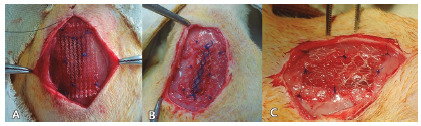
Meshes: A) Prolene®, B) Ultrapro® e C) Bard Soft®

A 1x2 cm defect was produced in the abdominal wall, preserving the integrity of the parietal peritoneum. The defect correction was performed using meshes with an area of ​​1.5x2.5 cm fixed in the extraperitoneal position, through four separate points of Prolene^®^ 5-0 fixing the angles in the aponeurosis of the abdominal wall 0.5 cm from the edge of the lesion, and four separate points interspersed with the first, fixing the mesh at the edges of the lesion (Figure 2). The skin was sutured with an intradermal stitch using 5-0 mononylon.

### Postoperative manipulation

Fragments of the wall were divided into a median section giving rise to a cranial and a caudal fragment. The segment containing mesh and musculature (cranial), without the skin, was subjected to tensiometric tests. The caudal fragments were kept in 10% formalin solution and used for microscopic analysis and the cranials placed in flasks with isotonic saline solution and kept in flasks with ice. For tensiometry an AG-I tensiometer (Shimadzu, Japan) was used with software Trapezium 2, where data on the area and tissue thickness and the results obtained were recorded. The tests were performed at a temperature of 24º C. The device was calibrated for a speed of 50 mm/min. The results were expressed in Newton (N). The cranial fragment was fixed to the tensiometer by the muscle tissues near the suture site.

### Microscopic analysis

H&E staining was used to analyze the inflammatory process, picrosirius red for types I and III collagens^13^. For the quantitative analysis of inflammatory parameters, the table by Vizzotto Junior^25^ was used.

In immunohistochemistry the ABCAN^®^ anti-MMP9 antibody (EP 1254) marker ab76003 was used. The TMA technique (tissue micro array) was used to make the block to be processed in the microtome, including the samples collected by the punch, representing the nine groups, and later making the slide^11^.

### Statistical analysis

The means between the results were submitted to and passed the KS normality test (Kolmogorov and Smirnov), suggesting parametric inference tests. From the unpaired, ANOVA test evidence of significant differences at the level of 5% probability between treatments in relation to the inflammatory reaction and analysis of collagens in the repetitions, was found, rejecting the null hypothesis at these observation points.

## RESULTS

### Tensiometry

**FIGURE 3:**
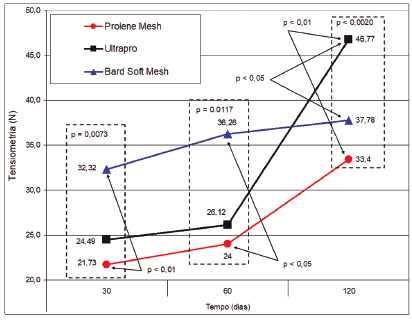
Comparison between groups in each period with emphasis on the significant differences in the tensiometric testing phases

A progressive increase in resistance to tension was observed in the Ultrapro^®^, highlighting its greater resistance in relation to the other two, and the Bard Soft^®^ maintained a level of stress rupture without significant increase since the beginning of the test. The mesh that showed the lowest tensile strength was the polypropylene microporous (Figure 3)^24^.

### Macroscopic evaluation

No animal presented hematoma, infection, fistula, suture dehiscence, incisional hernia, and the edges of the mesh fixation wounds were fully coaptized in all animals.

### Microscopic evaluation

#### Inflammatory process

**FIGURE 4:**
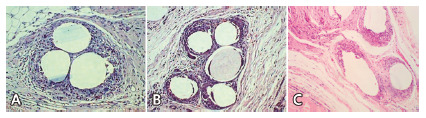
Results in 30 days: A) G1 30; B) G2 30; C) G330

Filamentous encapsulation was observed in the macroporous meshes and en-bloc in the microporous mesh (Figure 4).

Images A, B and C (Figure 4) show, respectively, block encapsulation on the Prolene^®^, filament encapsulation on the Ultrapro® and filament encapsulation on the Bard Soft^®^ meshes.

At 30 days, the inflammatory score showed a subacute inflammatory process in subgroups G1 30, G2 30, and G3 30. There was no statistically significant difference between the subgroups.

**FIGURE 5:**
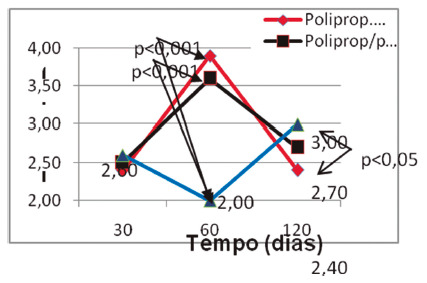
Comparison between groups in each period with emphasis on the significant differences in the phases of the inflammatory process

At 60 days, subgroups G1 60 and G2 60 showed an inflammatory process in the subacute phase, being more accentuated in subgroup G3 120, when compared to subgroup G1 120 (p <0.05, Figure 5).

#### Immunohistochemistry

In the individual analysis of the immunohistochemistry results with the MMP9 marker, it was found that in G1 there was a slight increase between 30 and 60 days (with no statistically significant difference), with a marked reduction between 60 and 120 days (p<0.05) In G2, the marker reduction was progressive from 30 to 120 days (p<0.05). G3 showed a reduction in the marker between 30 and 60 days, with a return to the initial levels at 120 days, with no statistically significant difference between periods.

**FIGURE 6:**
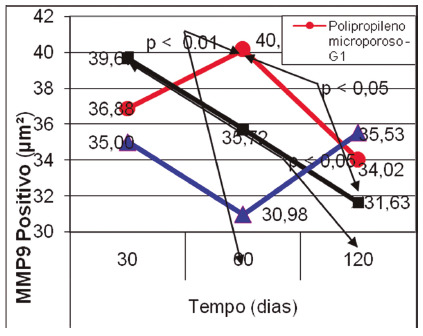
MMP9 comparison between groups in each period (30, 60 and 120 days) highlighting the significant differences

In the comparison between subgroups G1 60 and G3 30, the presence of the marker was more accentuated in subgroup G1 60 (p<0.01), where the inflammatory process was more accentuated (Figure 6).

#### Picrosirius red

**FIGURE 7:**
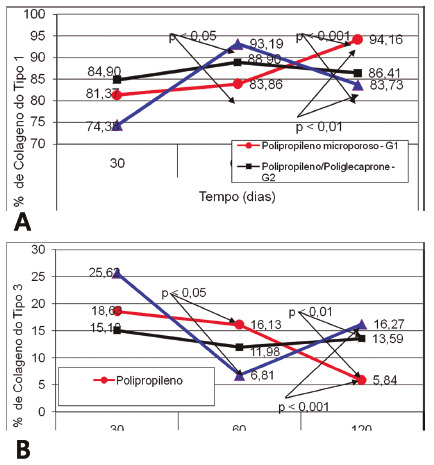
Results in collagen: A) significant type I in the comparison between groups in each period (30, 60 and 120 days); B) type III significant when comparing the groups in each period (30, 60 and 120 days)

Type I and type III collagens were analyzed. At 30 days in type I analysis, there was no statistically significant difference between groups. At 60 days, G1 showed a lower amount of type I collagen when compared to G3 (p<0.05), whereas at 120 days G1 had a greater amount of type I collagen compared to groups G2 and G3 (p<0.01, Figure 7). G1 showed a significant increase in the amount of type I collagen at 120 days compared to the first two observations 30 and 60 days. G3 showed a significant increase in the amount of type I collagen at 60 days compared to 30. At 60 days G1 showed a greater amount of type III collagen compared to G3 (p<0.05). At 120 days, groups G2 and G3 showed a greater amount of type III collagen compared to G1 (p<0.001). In the same period (120 days), G3 showed a slightly higher amount of type III collagen when compared to G2 (Figures 7A and B).

## DISCUSSION

### Surgical meshes

The evolution of polypropylene prostheses revolutionized surgery to correct abdominal wall defects. The creation of a low-weight prosthesis, in theory, induced less foreign body reaction, resulting in an improvement in the abdominal wall, with less contraction of the mesh and providing better incorporation of it in the abdominal wall^6, 9,15^.

Numerous modifications to the prosthesis designs have been investigated to reduce complications related to healing. Alteration in architecture to increase the pore area, low weight are the most important predictors of the performance of the biocompatibility of synthetic meshes. The ones with large pores show less inflammatory infiltrate, connective tissue and fibrotic bridges^27^.

The prostheses were chosen based on the widespread use of polypropylene today and considering that there are few studies comparing the low and high density mesh used in the extraperitoneal space in the period above 100 days.

Greca et al.^9^ showed in the comparison of low and high weight polypropylene meshes for the correction of abdominal wall defects in dogs including the peritoneum, incidence of 20% of seroma in both meshes, 5% of infection in high-weight prolene, 9.1% dehiscence in low weight and 4.6% in high weight meshes, and there was no incorporation in 5% of the high weight group. In this study, there was no hematoma, suture dehiscence, incisional hernia, wall infection, abscesses, fistulas or seroma.

In tensiometry, tissue rupture was always found outside the suture line, a result also obtained by Aydos et al.^1^, Pundek et al.^20^ and Utrabo CAL^23^ ^,^ ^24^. When comparing the meshes of subgroup G2 and G1, there was no significant difference, a coincident result with the Biondo-Simões et al.^4^ paper

It has already been reported that after the implantation of a high and low weight mesh, there was a significant increase in type I collagen in the mesh with larger and low weight pores^6^. A similar study evaluated the high-weight polypropylene mesh with pores smaller than 1 mm and the low-weight mesh with pores larger than 3 mm (Vypro^®^) with absorbable component. The wide-pored area was integrated with loose deposition of fibrosis interspersed with fatty tissue. On the contrary, with pores smaller than 1 mm, it was incorporated entirely with peripheral granulomas and scar tissue, forming bridges between the pores. It has been proven that the great distance between the filaments prevents the formation of these bridges.

The pore size of the mesh has an important influence on the biocompatibility of the foreign body after implantation.

The results of this study showed that despite fixation of the prosthesis with only four separate points in the aponeurosis and four interspersed fixing the prosthesis at the edge of the lesion, the incorporation was sufficient to avoid suture dehiscence. The results of this study also showed that at 120 days there is greater resistance of the wall corrected with the Ultrapro^®^ prosthesis, which has pores with a diameter greater than 3 mm² and a weight of approximately 28 g/m², in relation to the Prolene^®^, whose pores are less than 1 mm² in diameter. and an approximate weight of 100 g/m² and Bard Soft^®^, with a pore of approximately 6.29 mm², but with an approximate weight of 44 g/m². 

This increase in resistance is higher even when the resistance of the tissue is compared with the implantation of the Bard Soft^®^ prosthesis in 30 and 60 days, which has a pore with a larger diameter than the other study meshes, confirming the findings by Greca et al.^9^ and Pascual et al.^18^

Aydos et al.^1^ performing a tensiometer test only with the meshes, found that they had a greater breaking force than in the abdominal wall tissue treated with it and that the test in the tissue without the mesh the breaking tension was lower than that in the tissue mesh receiver.

This study showed that using a low weight, partially absorbable prosthesis, the result was satisfactory considering that the stress for the rupture of Ultrapro^®^ on the 30^th^ day has a value similar to the rupture stress of the Prolene^®^. Bard Soft® showed greater resistance at 60° day, despite the lower amount of polypropylene in the mesh in relation to subgroup G3, microporous. On the 120^th^ day, the wall corrected with Ultrapro^®^ showed greater resistance compared to the others.

It should be noted that Ultrapro^®^ and Bard Soft^®^ have pores with a diameter greater than 3 mm², respectively 3 to 4 mm² and 6.29 mm², however the weight of Bard Soft^®^ (44 g/m²) is higher than that of Ultrapro^®^ (28 g/m²) .

It is observed that Bard Soft^®^, despite having a pore with a larger diameter than Ultrapro^®^, presents a higher density due to the mesh design, as highlighted by Bellon^2^.

This study also demonstrated that the resistance of the mesh was adequate to correct the defect of the abdominal wall of the rat and maintain its integrity. White et al.^26^ described that the complete incorporation of the mesh in the recipient tissue is an important requirement to obtain solid repair. The degree of infiltration of the recipient tissue next to the biomaterial depends on the size of the pore when the prosthesis is incorporated into the recipient tissue, which is proportional to the degree of its porosity. The infiltration of fibrocytes and collagen from the recipient tissue in the prosthesis with adequate porosity occurs in approximately one month. Proper incorporation requires pores with sizes between 75 and 100 µm. The polypropylene monofilament mesh, with a pore greater than 100 µm, produces complete infiltration of the recipient tissue incorporating the entire prosthesis, as demonstrated in this study, which was also pointed out by others^6^ ^,^ ^14^ ^,^ ^18^. According to published reports, greater encapsulation occurs in the heavyweight meshes and consequent hardening of the corrected wall and better distribution of fibrosis among the filaments of the low-weight prosthesis, providing better elasticity and malleability of the corrected wall.

## CONCLUSIONS

The Ultrapro^®^ mesh showed better performance compared to the Prolene^®^ and Bard Soft^®^ in healing process of the abdominal wall.
